# Broadband Absorption and Efficient Hot-Carrier Photovoltaic Conversion based on Sunlight-induced Non-radiative Decay of Propagating Surface Plasmon Polaritons

**DOI:** 10.1038/s41598-017-05399-6

**Published:** 2017-07-06

**Authors:** Mengzhu Hu, Liu Yang, Hao Dai, Sailing He

**Affiliations:** 1 Centre for Optical and Electromagnetic Research, State Key Laboratory of Modern Optical Instrumentation, Zhejiang University, Hangzhou, 310058 P. R. China; 2 Department of Electromagnetic Engineering, JORCEP, School of Electrical Engineering, Royal Institute of Technology (KTH), S-100 44 Stockholm, Sweden

## Abstract

Localized surface plasmon polaritons (SPPs), which can decay non-radiatively into hot carriers, have been widely employed to extend the responses of traditional semiconductor-based photocatalytic and photovoltaic devices to sub-bandgap photons. However, radiative decay is unavoidable and adverse to device performances. Here, we propose to take advantage of propagating SPPs, another form of SPPs, which possess non-radiative decay only. A special gold-titanium dioxide nanowire array with each nanowire capped with a nanocone is proposed. The adjacent nanocones forming top gradual openings attribute to efficient sunlight harvesting, while the neighbouring nanowires forming bottom nanoslots allow sufficient absorption due to the propagating SPPs. With the combined advantages, almost 100% of light is absorbed by a very thin gold film in the visible range, and 73% in the whole considered range of 400–1170 nm, superior to the nanocone cell based on localized SPPs, let alone the nanowire-based and planar counterparts. Therefore, much better photovoltaic conversion performance is achieved with short-circuit current density of 0.74 mA/cm^2^ and open-circuit voltage of 0.41 V. This work confirms the superiority of non-radiative decay of propagating SPPs to the localized SPPs in terms of generation of hot carriers, providing a promising way of extracting electrons in metal into photocurrent.

## Introduction

Surface plasmon polaritons (SPPs) are strong interactions between the incident light and the surface electrons in metal, generating extremely strong electromagnetic field around the metal surface and thus possessing outstanding light trapping and manipulation properties^1^. The excited surface plasmons can decay either radiatively by re-emitting photons or non-radiatively through generation of energetic carriers (i.e., hot electrons and hot holes) via Landau damping^2–4^. Before thermal relaxation, these hot carriers can be extracted via plasmon-enhanced internal photoemission (IPE)^5, 6^ by e.g., contacting the metal with a semiconductor forming a metal-semiconductor Schottky junction^7–27^. In this process, the hot carriers are able to jump over the Schottky barrier at the metal-semiconductor interface as long as their energies are larger than the barrier height. Therefore, the photon energy to excite the hot carriers is not necessarily higher than the semiconductor bandgap. Since the Schottky barrier is usually lower than the semiconductor bandgap, incorporation of plasmonic metallic nanostructures is of great potential to extend the photoresponse of traditional pure semiconductor based photocatalytic and photovoltaic devices in a very broad wavelength range, thus attracting tremendous attention^2–4^.

Localized SPPs supported by gold (Au) nanoparticles have been widely investigated to enable titanium dioxide (TiO_2_) devices (bandgap: 3.0–3.2 eV) to respond to the visible and near-infrared photons^11–15^ and silicon devices (bandgap: 1.1 eV) to detect low-energy photons in the wavelength range of 1.2–2.5 μm^16^. The localized SPP resonances, as one form of SPPs, can be easily tuned by the nanoparticle size. However, they are always accompanied with radiative decay^28^, leading to less efficient light harvesting and thus lower power conversion efficiency (PCE). In order to suppress the radiation of localized SPPs and enhance their interactions with semiconductors, three-layer configurations of Au nanoparticles-TiO_2_-Au film have been reported with the bottom Au film serving as a reflective mirror^17–19^. Photonic crystal bandgap mode was also proposed to strengthen the localized SPPs of Au nanoparticles^20^. Recently, different plasmonic nanostructures have been developed to generate localized SPPs and enhance their non-radiative decay into hot carriers, e.g., vertical Au nanorod arrays coated with TiO_2_ for high-efficiency solar water splitting^21^ and as a solid-state photovoltaic solar cell^22^, large-area Au-TiO_2_ photonic crystal^23^ and Au nanograting on top of silicon^24^ for photodetection, etc. In contrast, propagating SPPs, as the other form of SPPs, cannot decay directly into photons and thus non-radiative decay is the only mechanism possible in flat films unless surface roughness is present^1, 2, 28^. Therefore, devices based on this mechanism are promising to generate greater hot carriers and thus higher PCE, in comparison with those based on localized SPPs. Planar plasmonic waveguides are usually employed to support propagating SPPs and demonstrate hot-carrier Schottky photodiode based on, e.g., metal stripes on top of a silicon film^25^ and a silicon waveguide^26^. In 2015, we proposed a special plasmonic waveguide by coating a silicon ridge waveguide with a thin Au film on its top and sidewalls and achieved greatly enhanced photoresponsivities over a very broad wavelength range from 1.2 to 1.6 μm^27^. However, they are in planar configurations and difficult for free-space light to couple in ref. 29. Therefore, solar photovoltaic and photocatalytic devices based on this mechanism have been seldom reported so far.

Here in this paper, we propose a new hot-carrier solar photovoltaic cell based on a distinct Au-TiO_2_ nanowire array with each nanowire capped with a nanocone. Nanoslots form between adjacent nanowires with gradually-increasing openings on the top. Here TiO_2_ is chosen as the semiconductor because it is very stable and has excellent electron-accepting ability^30^. In contact with Au, the Schottky barrier formed at the Au-TiO_2_ interface is about 1.07 eV^5^. In this structure, the sunlight can be efficiently coupled by the gradual openings into the nanoslots, where propagating SPPs are generated and almost totally decay into hot carriers. From the optical point of view, our design is a good broadband absorber. It is known that broadband sunlight absorption is the first step of any solar energy applications and is also one of the most important concerns for researchers in this field^31^. Taking advantage of the excellent light-trapping properties of both nanowire^32, 33^ and nanocone^34^ arrays, our design provides a distinctive alternative for efficient sunlight harvesting. It is similar to the previously-reported broadband absorbers based on pure Au^35, 36^ but here we demonstrate that only a very thin Au film coating on the TiO_2_ nanowires with their top nanocones is superior to those reported before in terms of both absorptance and bandwidth. Based on the broadband absorption, better photovoltaic conversion performance is thus predicted than the previously-reported solid-state plasmonic photovoltaics^22^.

## Results and Discussion

### Structure and Parameters

**Figure 1:**
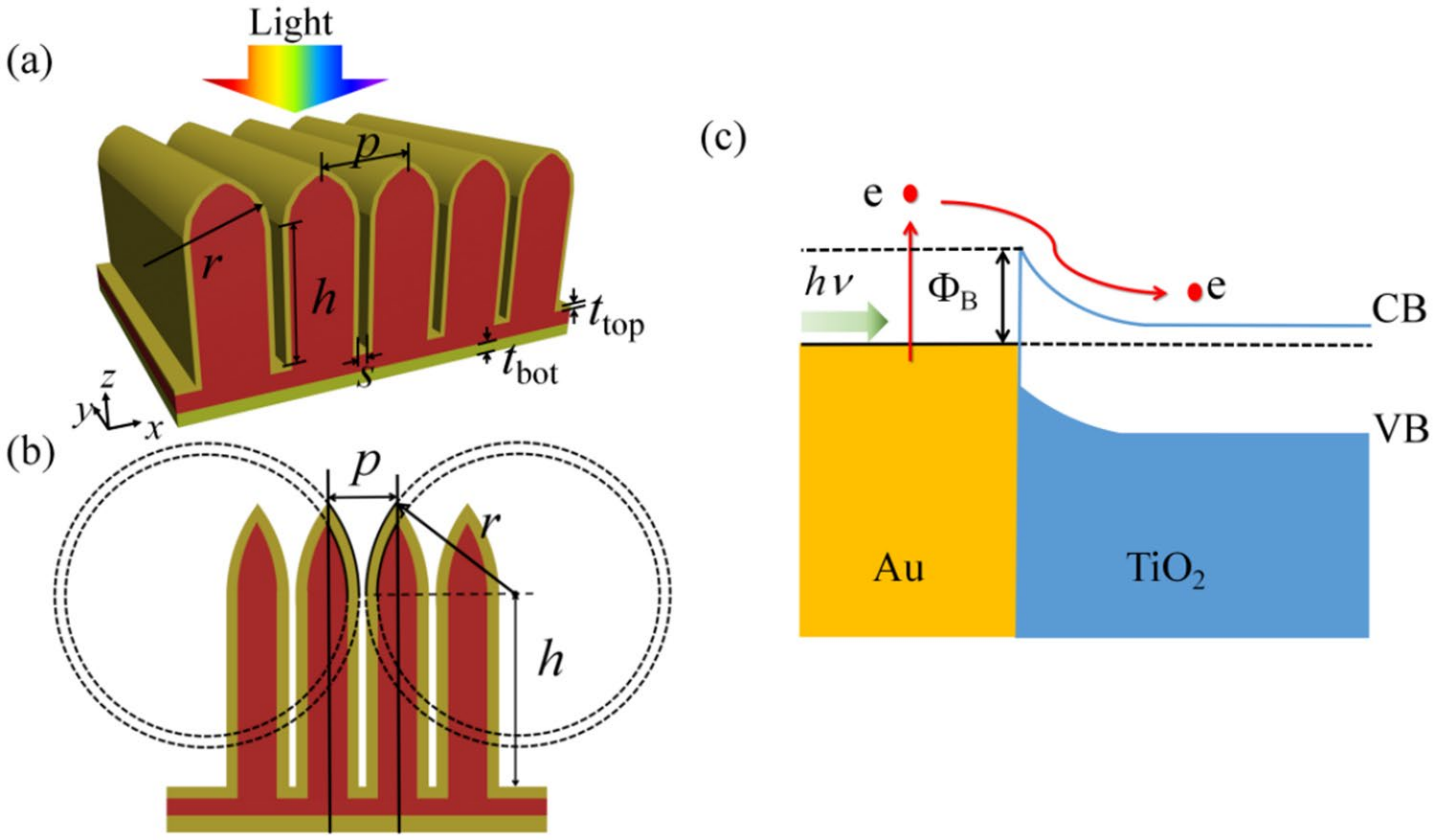
(**a**) Three-dimensional and (**b**) cross-sectional schematic diagrams of our proposed hot-carrier solar cell based on a special Au-TiO_2_ nanowire array with each nanowire capped with a nanocone; (**c**) Band alignment at the Au-TiO_2_ interfaces.

Our proposed hot-carrier solar cell is schematically shown in Fig. 1(a). It consists of two Au films and in between a TiO_2_ nanowire array (of period *p*) on top of a 100-nm thick TiO_2_ slab. Each TiO_2_ nanowire (of height *h*) is capped with a nanocone, which is formed by two intersectant circles with radii of *r*, as shown in Fig. 1(b). The top Au film of thickness *t*_top_ conformally covers the TiO_2_ nanowire array, forming narrow plasmonic nanoslots with spacing *s* between adjacent nanowires and the top gradually-increasing openings due to the nanocones. The sunlight illuminating from the top can be harvested and focused into the nanoslots through the gradual openings, which serve as bridges of gradually changing index from air to the nanoslots and allow high-efficiency sunlight harvesting with extremely low reflectivity. In particular for *p*-polarized light (with electric field along the *x* direction), the coupled light becomes vertically propagating SPP waves tightly confined and absorbed within the nanoslots. After going through the top Au film and the middle TiO_2_, only very weak light is able to reach the bottom 100-nm thick Au film and thereby less absorption can be obtained in the bottom. The light transmission through the bottom Au film with *t*_bot_ = 100 nm is also small. At both the top and bottom Au-TiO_2_ interfaces, Schottky contact forms and band bending occurs on the TiO_2_ side due to the different work functions, creating a barrier Φ_B_ = 1.07 eV^5^. The absorbed solar energy eventually decays into hot electrons. Those hot electrons with energies higher than Φ_B_ have certain probabilities to be extracted from Au into TiO_2_ via IPE^5, 6^, as demonstrated in Fig. 1(c). They form top and bottom photocurrents but have opposite flow directions. The net photocurrent is the difference of them and the direction is determined by the Schottky contact producing higher photocurrent at zero bias. In order to evaluate the device performance, both optical and electronic simulations are performed, which are described in detail in Methods.

### Broadband Absorption

**Figure 2:**
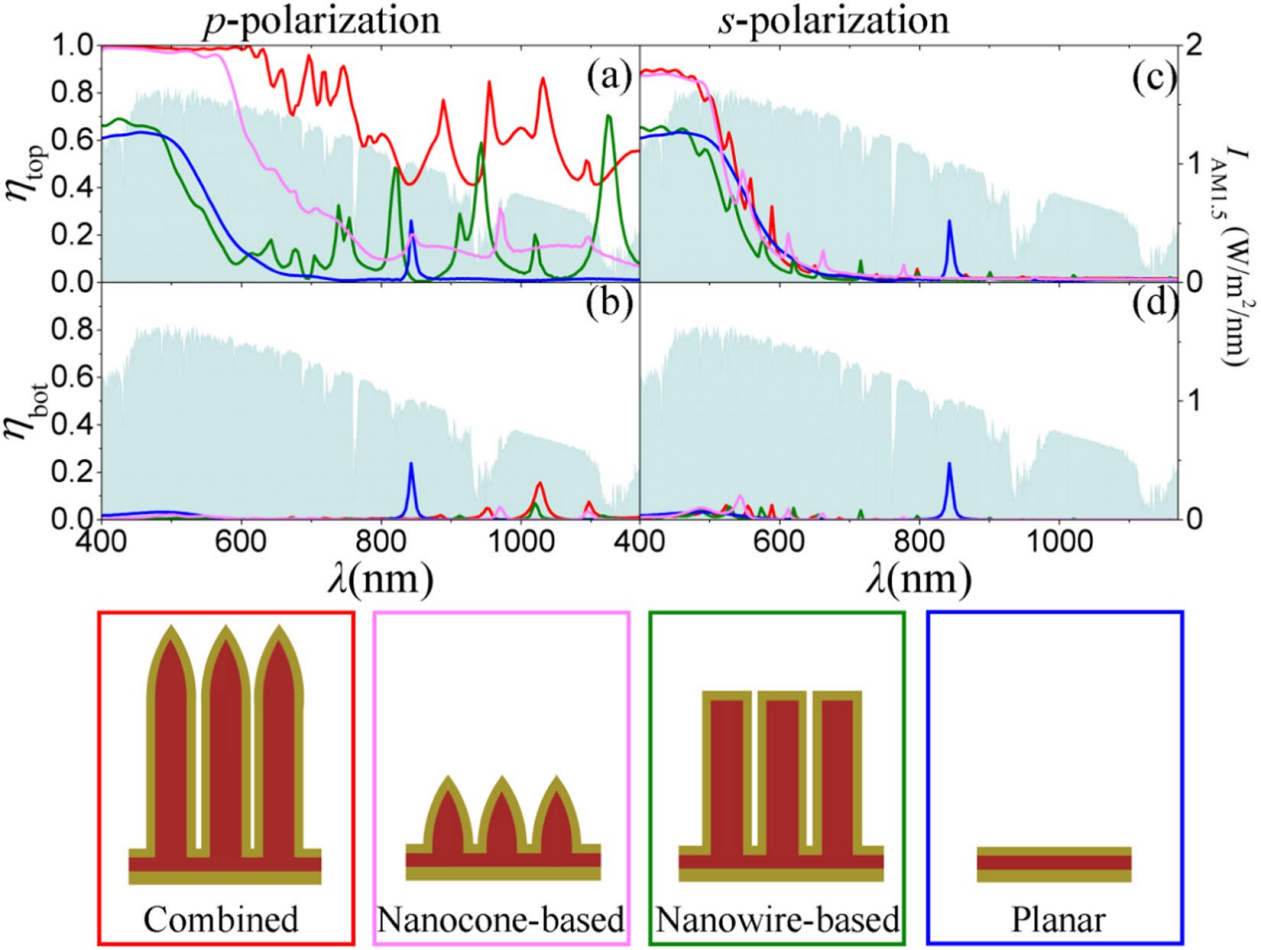
(**a**) Partial absorption spectra of (**a,c**) the top and (**b,d**) the bottom Au films in response to both *p*-polarized (**a,b**) and *s*-polarized (**c,d**) lights for our hot-carrier solar cell based on the combined structure of capped nanowires with top nanocones (red), the solar cells based on the nanocones only (pink) and the nanowires only (green), and the planar cell (blue), respectively. The solar spectral intensity is shown in all the four panels in light blue areas characterized by the right axes. The four solar cells involved in the comparison are schematically shown in the bottom inset. The geometrical parameters are *s* = 10 nm, *h* = 1 μm, *r* = 1 μm, *p* = 400 nm, and *t*_top_ = 70 nm.

Figure 2(a) and (b) show the partial absorption spectra of the top and bottom Au films, *η*_top_ and *η*_bot_, in response to the *p*-polarized light for our hot-carrier solar cell based on the capped nanowires with top nanocones (red), respectively. Those for the *s*-polarization are shown in Fig. 2(c) and (d). The geometrical parameters are *s* = 10 nm, *h* = 1 μm, *r* = 1 μm, *p* = 400 nm, and *t*_top_ = 70 nm. In order to clearly demonstrate the physical mechanisms and the performances of our design, other three structures are also investigated: one is based on the top nanocone array only without the bottom nanowires, another is based on the bottom nanowire array only without the top nanocones, and the other is the planar Au-TiO_2_-Au three-layer configuration, as schematically shown in the bottom inset of Fig. 2. Their geometrical parameters are kept the same as those of our proposed structure and their partial absorption spectra for both *p*- and *s*-polarizations are plotted in Fig. 2 for comparison. It is obvious that for both polarizations, *η*_top_ is much higher than *η*_bot_ over the whole wavelength range for all the four solar cells. Therefore, *η*_top_ dominates the net *J*_sc_ as discussed later.

As shown in Fig. 2(a), for *p*-polarization, the absorption spectrum in the top Au film for the traditional nanowire-based solar cell follows the one for the planar cell except several peaks in the long wavelength range beyond 600 nm, leading to higher absorption than the planar one. The similar spectra below 600 nm and the low baselines beyond 600 nm indicate that the nanoslots between neighbouring Au-TiO_2_ nanowires are too narrow to reduce the strong reflection of the flat Au film in the planar cell. By the pure nanocone array, the strong reflection is greatly suppressed. Absorption as high as 100% is observed in Fig. 2(a) in the wavelength range below 561.7 nm. Beyond 561.7 nm, the absorption drops quickly but is still higher than that of the planar cell, illustrating that the gradually-increasing openings formed by the adjacent nanocones are favorable for sunlight harvesting. Most interestingly, by capping each nanowrie with a nanocone, significant improvement in absorption is achieved over the whole solar spectrum. Especially the 100% absorption band extends to about 650 nm. Due to the ripples and the apparent absorption peaks, the long-wavelength absorption is obviously higher than those for the cells based on the pure nanowire and pure nanocone arrays, as shown in Fig. 2(a). For the *s*-polarization shown in Fig. 2(c), all the four absorption spectra illustrate again the superior light coupling ability of the nanocones (either combined with or without bottom nanowires) to the others without nanocones. Many ripples induced by Fabry-Parot resonances appear on the spectra for all the solar cells except the planar one, but their average absorptions are still lower than those for the *p*-polarization. This indicates that some SPP modes special for the *p*-polarization must be excited, leading to stronger light confinement and higher absorption than those for the *s*-polarization.

**Figure 3:**
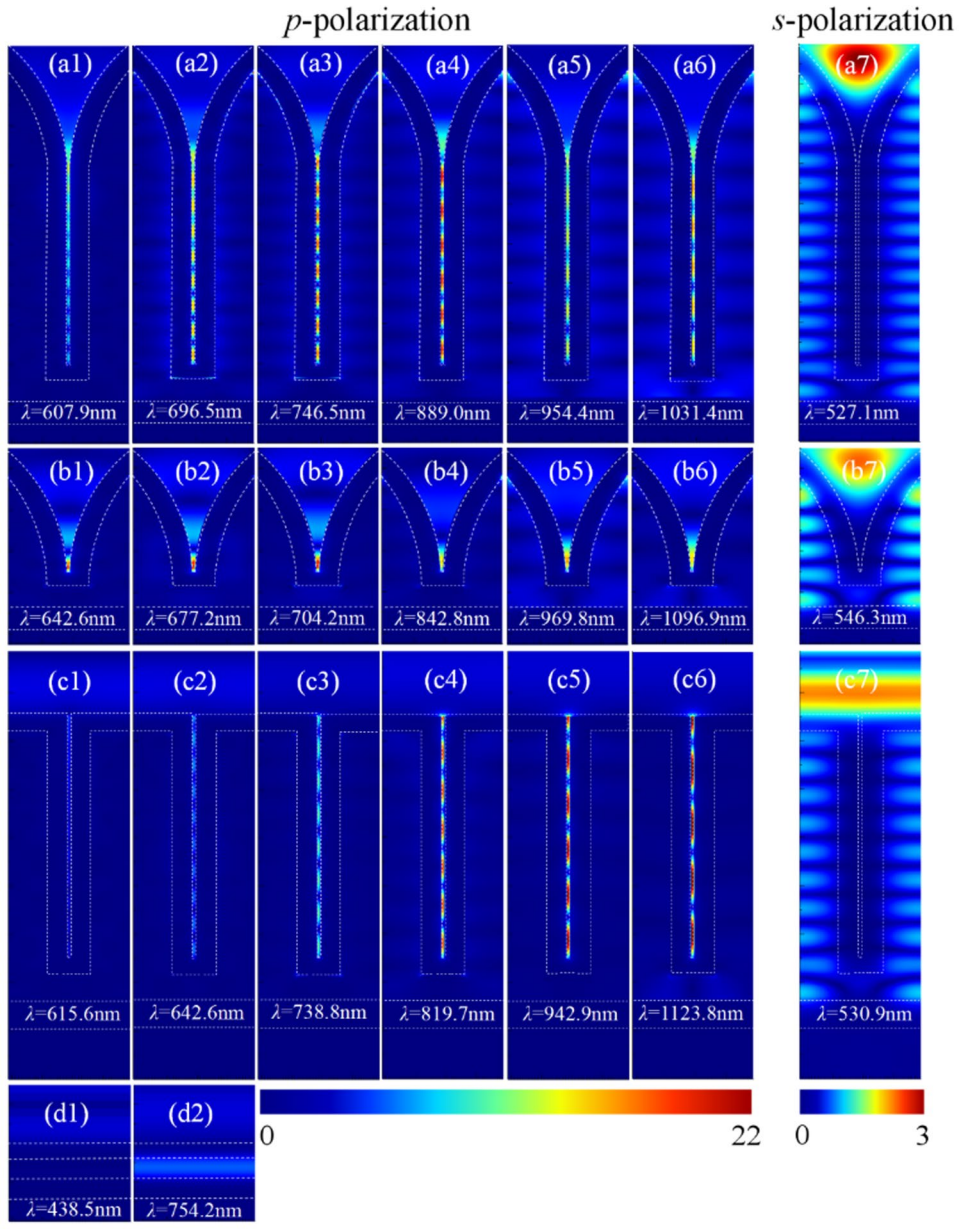
Distributions of the amplitude of electric field for *p*-polarization at some representative absorption peak wavelengths for our hot-carrier solar cell based on the combined structure of capped nanowires with top nanocones (the first row): (**a1**) *λ* = 607.9 nm, (**a2**) *λ* = 696.5 nm, (**a3**) *λ* = 746.5 nm, (**a4**) *λ* = 889.0 nm, (**a5**) *λ* = 954.4 nm, (**a6**) *λ* = 1031.4 nm; the solar cell based on the nanocones only (the second row): (**b1**) *λ* = 642.6 nm, (**b2**) *λ* = 677.2 nm, (**a3**) *λ* = 704.2 nm, (**a4**) *λ* = 842.8 nm, (**a5**) *λ* = 969.8 nm, (**a6**) *λ* = 1096.9 nm; the solar cell based on the nanowires only (the third row): (**c1**) *λ* = 615.6 nm, (**c2**) *λ* = 642.6 nm, (**c3**) *λ* = 738.8 nm, (**c4**) *λ* = 819.7 nm, (**c5**) *λ* = 942.9 nm, (**c6**) *λ* = 1123.8 nm; and the planar cell (the bottom row): (**d1**) *λ* = 438.5 nm, (**d2**) *λ* = 754.2 nm. Distributions of the amplitude of electric field for *s*-polarization (**a7**) at *λ* = 527.1 nm for our hot-carrier solar cell, (**b7**) at *λ* = 546.3 nm for the cell based on the nanocones only, and (**c7**) at *λ* = 530.9 nm for the cell based on the nanowires only, respectively.

Figure 3 shows the distributions of the amplitude of electric field at some representative absorption peak wavelengths for both polarizations and for all the four structures schematically shown in Fig. 1 and the inset of Fig. 2. For the planar solar cell, the top Au film is a good reflector due to its large imaginary parts of the dielectric constants. As illustrated in the electric field distributions in Fig. 3(d1) and (d2), SPPs cannot be excited. The high absorption of about 0.6 below 500 nm (Fig. 2(a)) is mainly attributed to the interband transitions in the top Au film. Without the interband transitions beyond 500 nm, the absorption drops quickly. At *λ* = 754.2 nm, constructive interference of the light between the two Au films happens as illustrated in Fig. 3(d2), leading to a small absorption peak for both the top and bottom Au films (Fig. 2(a)). Due to the geometric symmetry, the absorption curve and the field distributions are all the same for the orthogonally polarized light.

**Figure 4:**
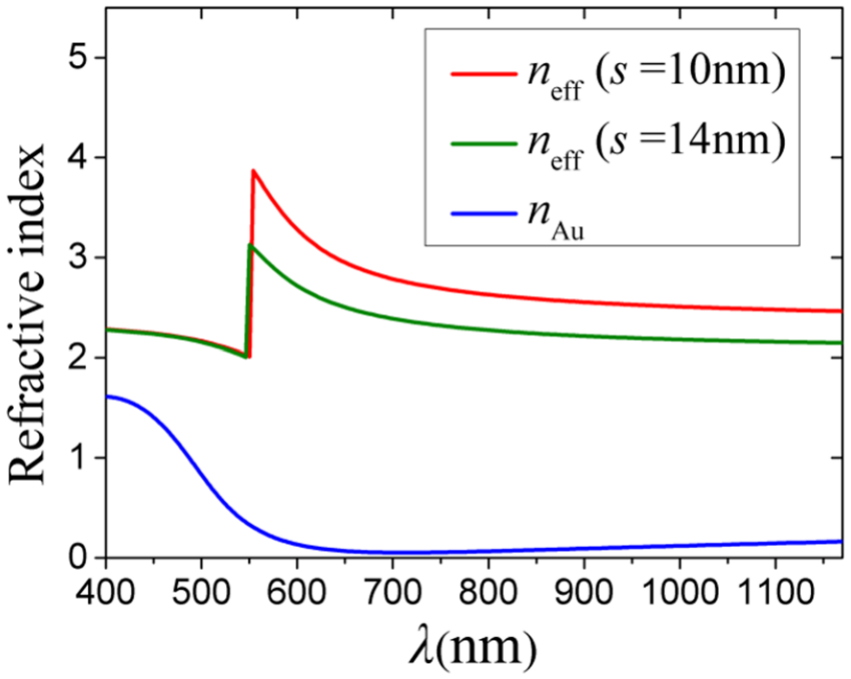
Effective refractive indices (n_eff_) of fundamental SPP mode of a TiO_2_-Au-air-Au-TiO_2_ multilayer plasmonic waveguide with air spacing, *s* = 10 (red) and 14 (green) nm. The real parts of the refractive index of Au (n_Au_)^37^ is also plotted as a blue curve.

The light reflection is not mitigated by introducing nanoslots in the top Au film. Even at the absorption peak wavelengths, where strong light resonance and confinement occur, reflection can still be seen from the electric field distributions for both polarizations in Fig. 3(c1–7). Actually, the nanoslots can be seen as a TiO_2_-Au-air-Au-TiO_2_ multilayer plasmonic waveguide array, which support SPP waves for *p*-polarization. Such waveguides always have very high effective refractive indices, n_eff_, in contrast to air, which is shown in Fig. 4. It is difficult for light coupling. However, once the free-space light is coupled into the nanoslots, SPP waves are excited propagating along the nanoslots. Due to the reflections at the end of the plasmonic waveguides with limited lengths, the SPP waves are bounced back and forth within them, leading to apparent interference resonances as shown in Fig. 3(c1–6). Due to the non-radiative decay of the propagating SPPs, at each constructive interference, there is an absorption peak appearing in the absorption spectrum, as shown in Fig. 2(a). Looking closer at Fig. 3(c1–6), it is found that the period of the interference fringes corresponds to half of the SPP effective wavelengths (=*λ*/n_eff_), confirming again that fundamental SPP modes are excited in the nanoslots. In great contrast, for the *s*-polarization, SPPs are not supported and no optical field is localized in the nanoslots, as demonstrated in Fig. 3(c7). Most light is reflected, leaving little transmitting through the top 70-nm thick Au film and resonating in TiO_2_. Due to various orders of Fabry-Parot resonances, multiple small absorption peaks appear on the absorption spectrum, as shown in Fig. 2(c).

For the pure nanocone array, for *p*-polarization, the gradually-increasing openings can be seen as TiO_2_-Au-air-Au-TiO_2_ multilayer plasmonic waveguides with varying air widths. The coupled light is converted into SPPs propagating along the Au sidewalls of the openings. Since n_eff_ increases as the air width decreases, as demonstrated in Fig. 4, the velocity of the propagating SPPs becomes slower toward the bottom, where they are finally localized (stopped with zero group velocity), as shown in Fig. 3(b1–6). Such localized SPPs always radiate photons, leading to weak non-radiative decay in the long wavelength range of *λ* > 561.7 nm in Fig. 2(a). Since the intrinsic absorption of Au is very high in the range of *λ* < 561.7 nm, the coupled light can be almost totally absorbed with little reflection. For *s*-polarization, SPPs disappear in the gradual openings and Fabry-Parot resonances in the TiO_2_ appear, as demonstrated in Fig. 3(b7), leading to small absorption peaks in Fig. 2(c). The high absorption in the short wavelength range also benefits from the gradually-increasing openings, which help light coupling. Nevertheless, the gradual openings with the largest width of *p* = 400 nm are too small for photons of *λ* > 650 nm to couple in. Therefore, almost no absorption is seen in this wavelength range in Fig. 2(c).

By capping each nanowrie with a nanocone, we achieve both desired properties of the top gradually-increasing openings and the bottom nanoslots. For *p*-polarization, propagating SPPs excited in the gradual openings do not stop but go into the nanoslots, leading to strong SPP resonances within the nanoslots (Fig. 3(a1–6)), similar to the behaviors in the nanowire-based cell (Fig. 3(c1–6)). With the combined advantages of the top gradual openings and the bottom nanoslots, much higher absorption induced by the efficient light coupling and the non-radiative decay of SPPs is achieved over the whole wavelength range compared with the cell based on the nanocones only or the nanowires only, as shown in Fig. 2(a). For *s*-polarization, SPPs cannot be excited and the top gradual openings play a dominant role in light coupling and absorption. Therefore, the absorption spectrum has almost the same trend as that for the nanocone-based cell (Fig. 2(c)). More absorption peaks appear because of the extended TiO_2_ nanowire, where more resonances appear as demonstrated in Fig. 3(a7). From the above analysis, it is clear that there are two important mechanisms embedded in our hot-carrier solar cell for the broadband absorption covering the main solar spectrum: (i) the gradually-increasing openings formed by the top nanocones greatly minimizes the light reflection and enhances light harvesting; (ii) propagating SPP waves generated specially by the coupled *p*-polarized light resonate within the nanoslots, leading to sufficient absorption by the Au sidewalls.

**Figure 5:**
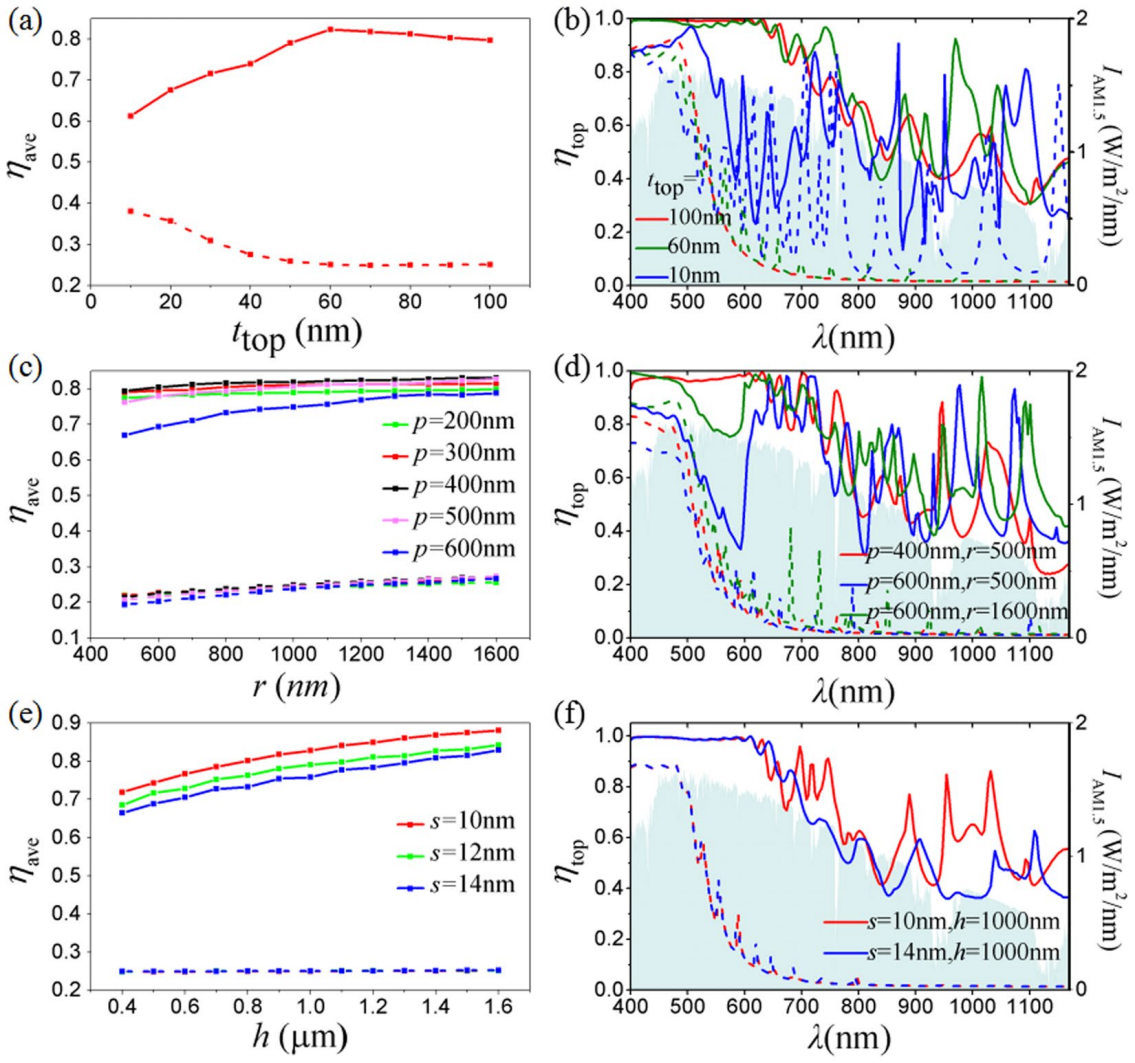
Averaged absorption, *η*_ave_, of our hot-carrier solar cell based on the combined structure of capped nanowires with top nanocones for both *p*-polarization (solid curves) and *s*-polarization (dotted curves) as a function of (**a**) the top Au film thickness, *t*_top_, with *s* = 10 nm, *h* = 1 μm, *r* = 1 μm, and *p* = 400 nm; (**c**) the circle radius to form the nanocones, *r*, for different period, *p*, with *s* = 10 nm, *h* = 1 μm, and *t*_top_ = 70 nm; (**e**) the nanoslot height, *h*, for different nanoslot widths, *s*, with *r* = 1 μm, *p* = 400 nm, and *t*_top_ = 70 nm. Some representative absorption spectra in the top Au film, *η*_top_, for both *p*-polarization (solid curves) and *s*-polarization (dotted curves) are plotted in Fig. 5(b,d,f) corresponding to *η*_ave_ in Fig. 5(a,c,e), respectively. In Fig. 5(b,d,f), the solar spectral intensity is shown in all the four panels in light blue areas characterized by the right axes.

To further illustrate the physical mechanisms, the geometric effects of our hot-carrier solar cell on the average absorption, *η*_ave_, are investigated for both polarizations and demonstrated in Fig. 5. Here, only the absorption in the top Au film is considered because it dominates the total absorption for both polarizations as mentioned above. *η*_ave_ is calculated by integrating the absorption spectrum weighted with the solar spectral intensity to evaluate the sunlight harvesting ability. From Fig. 5(a), it is seen that *η*_ave_ first increases and then decreases slowly with the increasing thickness of the top Au film, *t*_top_, for *p*-polarization. It peaks at *t*_top_ = 60 nm with *η*_ave_ ≈ 82.3%. For thinner Au film, e.g., *t*_top_ = 10 nm, most light is transmitted through with greatly reduced absorption over the whole wavelength range in comparison with that for the 60-nm thick Au film (Fig. 5(b)). For even thicker Au films, whose intrinsic absorption is very high, less light can be involved in the resonances in the nanoslots. Therefore, its absorption is a little stronger below 650 nm but weaker beyond it, as indicated by the absorption spectrum of the 100-nm thick Au film in Fig. 5(b). For *s*-polarization, *η*_ave_ is much lower than that for the *p*-polarization (Fig. 5(a)). As *t*_top_ increases, light transmitting into TiO_2_ becomes increasingly small until *t*_top_ = 60 nm, where the light transmission is blocked. With weaker Fabry-Parot resonances in TiO_2_, the resonance-induced absorption in the top Au film becomes weaker too (as demonstrated in Fig. 5(b)). Therefore, *η*_ave_ decreases gradually until *t*_top_ = 60 nm, where *η*_ave_ starts to keep unvaried (Fig. 5(a)).

As shown in Fig. 1(b), the nanocones on top of the nanowires are characterized by the period, *p*, and the circle radius, *r*. Period *p*, which also characterizes the largest width of the gradual opening, affects how much light can be received, while *r* determines the index change rate and thus the light reflectivity. A balance of these two opposite effects can give rise to the largest *η*_ave_. If *p* is small, i.e., *p* < 500 nm, the absorption mainly depends on the received light instead of the reflected light. Therefore, as *p* increases, *η*_ave_ for the *p*-polarization rises but appears to be independent of *r*, as shown in Fig. 5(c). When *p* ≥ 500 nm, *η*_ave_ becomes sensitive to *r*, rising with the increasing *r*. In this case, for a given *p*, if *r* becomes larger, the index change rate as well as the induced light reflectivity become smaller, which can be seen in Fig. 5(d). Thus *η*_ave_ increases. Unfortunately, for all the considered *r*-values here, *η*_ave_ drops when *p* is larger, because the negative effect of the light reflectivity surpass the positive effect of the received light. Since these two effects are not sensitive to the light polarization, the curves in Fig. 5(c) for *s*-polarization follow similar trends for *p*-polarization.

For *s*-polarization, no optical field can be confined in the nanoslots, as shown in Fig. 3(a7). Therefore, *η*_ave_ is insensitive to the geometric parameters of the nanoslots, i.e., the height, *h*, and the spacing, *s* (Fig. 5(e)). For *p*-polarization, if *h* becomes larger, more SPP resonances can be generated and *η*_ave_ must rise. For a give *h*, when *s* becomes smaller, n_eff_ of the SPP mode increases (Fig. 4), leading to more resonances in the range of *λ* > 600 nm (Fig. 5f). Thus, *η*_ave_ rises with the decreasing *s* (Fig. 5(e)).

### Photovoltaic Conversion

**Figure 6:**
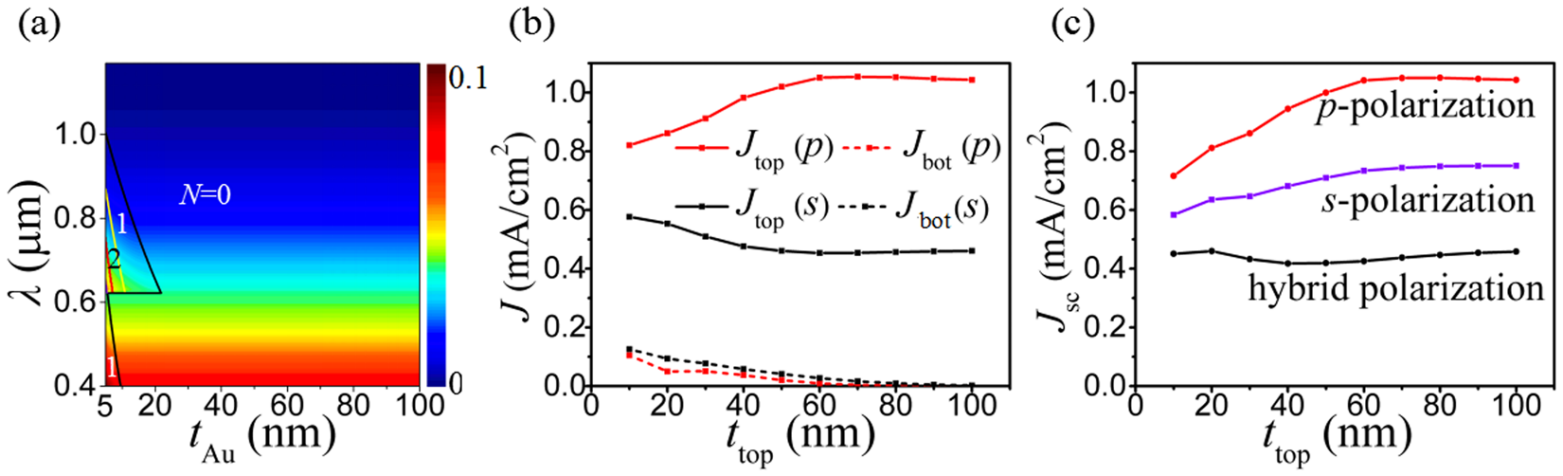
(**a**) IQE of a Au-TiO_2_ Schottky diode as a function of Au film thickness, *t*_Au_, and the photon wavelength, *λ*. The whole *λ*-*t*_Au_ area is divided by three curves into different regions where the hot electrons can transport *N* ( = 0, 1, 2, 3) round trips within the Au film. The *N* = 3 region is very small on the left side of the red curve. (**b**) The photocurrent density generated in the top Au film, *J*_top_ (solid curves), and in the bottom Au film, *J*_bot_ (dotted curves), for both *p*-polarization (red), *s*-polarization (black) as a function of the top Au film thickness, *t*_top_. (**c**) The net photocurrent density, *J*_sc_, for both *p*-polarization (red), *s*-polarization (black) and hybrid polarization (purple) as a function of *t*_top_. The other geometrical parameters are *s* = 10 nm, *h* = 1 μm, *r* = 1 μm, and *p* = 400 nm.

Figure 6(a) shows internal quantum efficiency (IQE) of an Au-TiO_2_ Schottky diode with different Au thicknesses, *t*_Au_. Since IQE is greatly dependent on the number of round trips, *N*, of a hot carrier traveling within the Au film (Methods), three curves are plotted and overlaid on Fig. 6(a), dividing the whole *λ*-*t*_Au_ area into four regions with *N* = 0, 1, 2, and 3. From this figure, it is seen that *N* = 0 takes up most of the *λ*-*t*_Au_ area (on the right side of the black curve), meaning that the excited hot electrons has only one chance to jump over the Schottky barrier and contribute to the photocurrent. Therefore, IQE is independent of *t*_Au_ but it decreases as the photon energy decreases (correspondingly *λ* increases). On the left side, *t*_Au_ becomes smaller than MFP and the hot electrons can gain more probabilities for IPE with increasing *N*. IQE increases. For the hot electrons gained energies from the long-wavelength photons (e.g., *λ* > 624 nm), MFP = 70 nm is large enough to allow maximal round trips of *N* = 3 (on the left side of the red curve in Fig. 6(a)). However, in the range of *λ* < 624 nm, due to the small MFP (=18 nm) induced by the interband transitions, maximal *N* = 1 is allowed in the lower left corner. Despite of this, because of the higher photon energy, IQE in this region is still higher than that in the above regions with *N* = 1, 2, and 3, as shown in Fig. 6(a).

With IQE shown in Fig. 6(a), the photocurrent densities (*J*_top_, *J*_bot_, as well as *J*_sc_) are easily calculated by Equation (1). For the bottom Au-TiO_2_ contact, IQE is fixed at *t*_bot_ = *t*_Au_ = 100 nm and therefore *J*_bot_ follows the trend of the absorption in the bottom Au film, *η*_bot_, which decreases as the top absorption, *η*_top_, increases, and vice versa. For the top Au-TiO_2_ contact, IQE changes only with the Au film thickness, *t*_top_, among all the structural parameters. The effects of other parameters on *J*_top_ follow the same trends of their effects on *η*_ave_, as demonstrated in Fig. 5(c) and (e). *J*_top_ will become even larger than *J*_bot_ if *η*_top_ is enhanced. Therefore, it is easy to understand that *J*_sc_ behaves similarly as *η*_ave_ when *r*, *p*, *h*, *s* vary besides larger variation extents for both polarizations.

As shown in Fig. 6(b), *t*_top_, on which both *η*_top_ and IQE depend, influences *J*_top_ directly (according to Equation (2) in Methods), while *J*_bot_ is affected by *t*_top_ indirectly through *η*_top_ and behaves oppositely as compared to *η*_ave_ with *t*_top_ as shown in Fig. 5(a). When *t*_top_ > 30 nm, IQE keeps unvaried and *J*_top_ follows the same trend as *η*_ave_ as demonstrated in Figs 5(a) and 6(b). For *p*-polarization, *J*_top_ increases (*J*_bot_ decreases) as *t*_top_ increases until *t*_top_ = 60 nm where *J*_top_ keeps almost unvaried. The decreasing *J*_bot_ equivalently exerts an up-rising tendency to *J*_top_, causing a quicker rising rate for *J*_sc_ before *t*_top_ = 60 nm as demonstrated in Fig. 6(c). In Fig. 6(b), *J*_top_ for *s*-polarization decreases until *t*_top_ rises to 60 nm and then remains unchanged. But *J*_bot_ continues to decrease slowly as *t*_top_ increases from 30 nm to 100 nm. Therefore, *J*_sc_ for *s*-polarization first decreases and then increases (Fig. 6(c)). On the other hand, when *t*_top_ < 30 nm and increases in this range, IQE decreases drastically. In this case, *J*_top_ for *p*-polarization in Fig. 6(b) does not increase as quickly as *η*_ave_ shown in Fig. 5(a). Because of the decreasing *J*_bot_, *J*_sc_ for the *p*-polarization rises as shown in Fig. 6(c). For *s*-polarization, both *J*_top_ and *J*_bot_ decrease as *t*_top_ increases. The different decreasing rates result in a maximum of *J*_sc_ at *t*_top_ = 20 nm (Fig. 6(c)). Generally speaking, due to the non-radiative decay of SPPs as analyzed before, *J*_sc_ for *p*-polarization is much higher than that for *s*-polarization. *J*_sc_ for the hybrid polarization lies between the two curves. As *t*_top_ increases, *J*_sc_ quickly increases to *J*_sc_ = 0.74 mA/cm^2^ at *t*_top_ = 70 nm and then rises at a slower rate (Fig. 6(c)).

**Figure 7:**
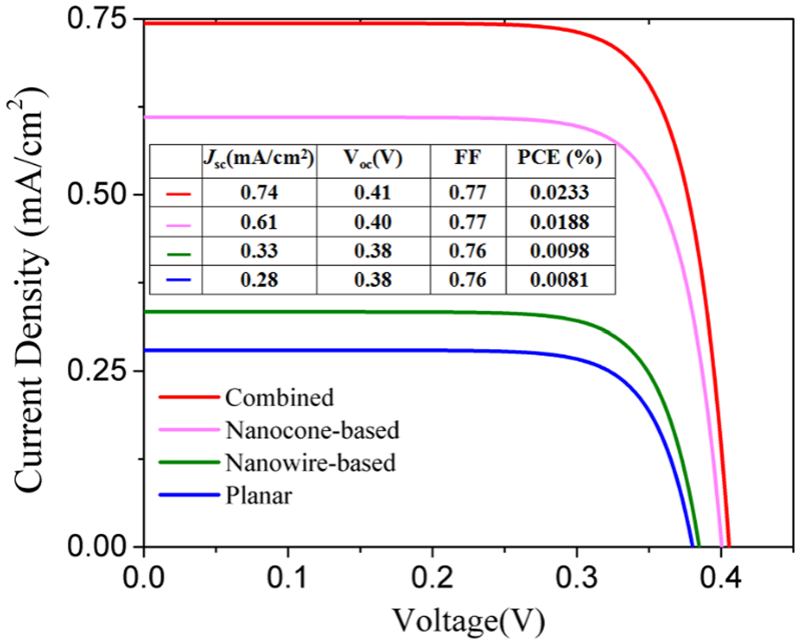
*J*-*V* curves for our hot-carrier solar cell based on the combined structure of capped nanowires with top nanocones (red), the solar cells based on the nanocones only (pink) and the nanowires only (green), and the planar cell (blue). The inset table summarizes the characteristic parameters, i.e., *J*_sc_, *V*_oc_, FF, of the four cells. The geometrical parameters are *s* = 10 nm, *h* = 1 μm, *r* = 1 μm, *p* = 400 nm, and *t*_top_ = 70 nm.

Due to the broadband absorption achieved by the capped nanowires with top nanocones, our hot-carrier solar cell is superior to the other three comparative cells in terms of short-circuit current density, *J*_sc_, open-circuit voltage, *V*_oc_, fill factor, FF, and PCE (Methods), as shown in Fig. 7. The four *J*-*V* curves have similar FF values and therefore, *J*_sc_ and *V*_oc_ dominate PCE. *J*_sc_ of our cell is more than 2.2 (2.6) times of that of the nanowire-based solar cell (the planar one) mainly due to the better sunlight coupling. *V*_oc_ is also greater because of the higher *J*_sc_ according to the following equation derived from Equation (7) in Methods,

1 $${V}_{{\rm{oc}}}=\frac{kT}{q}\,\mathrm{ln}(\frac{{J}_{sc}}{A\ast {T}^{2}{e}^{-{{\rm{\Phi }}}_{{\rm{B}}}/kT}}+1)$$

In comparison with the nanocone-based solar cell, which depends on localized SPPs, our solar cell based on propagating SPPs has higher *J*_sc_ and conseqently *V*_oc_, as shown in Fig. 7, indicating again the more effectiveness of non-radiative decay of propagating SPPs than the localized SPPs. As a result of the enhanced *J*_sc_ and *V*_oc_, PCE is also improved. These characteristic parameters listed in the inset of Fig. 7, which are all much better than those for a solid-state hot-carrier solar cell reported in ref. 22, can be further improved by minimizing the polarization sensitivity. Due to the low IQE (<0.1 in Fig. 6(a)), PCE is extremely low, which however could be increased to >20% by engineering the electron density of sates of the absorber^6^.

## Conclusion

We have proposed an efficient hot-carrier photovoltaic solar cell based on the sunlight-induced non-radiative decay of propagating SPPs. It consists of a special Au-TiO_2_ nanowire array with each nanowire capped with a nanocone. The top gradually-increasing openings formed by the adjacent nanocones bridges the air and the high-n_eff_ nanoslots formed by the neighbouring nanowires, leading to excellent sunlight harvesting and effective excitation of propagating SPPs specially for the *p*-polarization. Therefore, broadband absorption is achieved. Specifically, almost 100% of light is absorbed by the top Au film in the range of 400–650 nm, about 93% in the range of 400–800 nm and 73% in the whole considered range of 400–1170 nm, superior to those reported before in terms of both absorptance and bandwidth^35, 36^. Because the propagating SPPs (rather than the localized SPPs) can almost totally decay into hot carriers^1, 2, 28^, better photovoltaic conversion performance is achieved in terms of *J*_sc_, *V*_oc_, and PCE compared with that of the nanocone-based solar cell, where localized SPPs are the main mechanism for the high absorption, let alone the performances of the nanowire-based cell and the planar cell. The characteristics are also superior to the previously-reported solid-state plasmonic photovolltaic device^22^. Much higher PCE can be achieved by e.g., eliminating the polarization sensitivity, engineering the electron density of states of Au^6^, etc., providing an efficient way of converting metallic absorption into photocurrent by extracting electrons in metal.

## Methods

### Optical Simulation

Two-dimensional full-wave numerical simulation is performed based on a finite-difference time-domain (FDTD) method (Lumerical FDTD Solutions). Plane wave is incident normally from the top. Since hot-carrier devices only respond to photons with higher energies than Φ_B_ = 1.07 eV^5^, the incident light wavelength *λ* is set covering the main solar spectrum peak ranging from 400 nm to a Φ_B_-determined value, i.e., 1170 nm. In this wavelength range, TiO_2_ is transparent^30^. Both *p*-polarization and *s*-polarization (with electric field along the *x* and *y* direction, respectively) are considered. Periodic boundaries are set in the *x* direction and perfectly-matched layer boundary treatment is used in the *z* direction. The refractive index of TiO_2_ is set to 2.43 because it changes little in our considered wavelength range, while the refractive indices of Au are obtained from ref. 37. With this model, partial absorptions in both the top and bottom Au films, *η*_top_(*λ*) and *η*_bot_(*λ*), can be obtained by integrating the square of electric field intensity with the imaginary part of the dielectric constants of Au over different areas and normalized by the incident light power, as derived previously in ref. 38.

### Electronic Model for Internal Photoemission

Based on *η*_top_(*λ*) and *η*_bot_(*λ*), the top and bottom photocurrent densities can be obtained by ref. 38:

2 $${J}_{{\rm{top}}{\rm{or}}{\rm{bot}}}=q{\int }_{400\,{\rm{nm}}}^{{\rm{1170}}\,{\rm{nm}}}{\phi }_{{\rm{AM1}}{\rm{.5}}}(\lambda )\cdot {\eta }_{{\rm{top}}{\rm{or}}{\rm{bot}}}(\lambda )\cdot {{\rm{IQE}}}_{{\rm{top}}{\rm{or}}{\rm{bot}}}(\lambda )d\lambda ,$$

where *q* is the elementary charge, *φ*_AM1.5_(*λ*) is photon flux of the incident sunlight light, i.e., *φ*_AM1.5_(*λ*) = *I*_AM1.5_(*λ*)/(*hν*), *I*_AM1.5_(*λ*) is the AM 1.5 solar spectral irradiance, as shown in Figs 2 and 5, *h* is Planck’s constant, *ν* = c/*λ* is the light frequency, and IQE(*λ*) is internal quantum efficiency (IQE), which determines how many hot electrons can jump over the Schottky barrier. It can be estimated by integrating the IPE probabilities, *P*(*E*), of hot electrons in the energy range from Φ_B_ to the initial excess energy, *E*_0_, obtained from the absorbed photons, as expressed below:

3 $${\rm{IQE}}(\lambda )=\frac{1}{{E}_{0}}{\int }_{{{\rm{\Phi }}}_{{\rm{B}}}}^{{E}_{0}}P(E)dE.$$

According to our previous model for IPE reported in ref. 27, at a semi-infinite Au-TiO_2_ interface, *P*(*E*) is calculated based on a momentum cone with its solid angle, Ω, determined by $\cos \,\Omega =\sqrt{{{\rm{\Phi }}}_{{\rm{B}}}/E}$, within which only hot electrons with energies higher than the Schottky barrier are able to be extracted from Au to TiO_2_. Those that cannot jump over the barrier are assumed to be reflected back elastically. In this case, one hot electron has only one opportunity for emission and *P*(*E*) can be expressed as:

4 $$P(E)=0.5(1-\,\cos \,{\rm{\Omega }})=0.5(1-\sqrt{{{\rm{\Phi }}}_{{\rm{B}}}/E}).$$

However, for cases considered in this work, the Au film has a certain thickness as shown in Fig. 1. The situation for the movement of hot electrons becomes complicated. Assuming that the electrons which cannot transmit through the Au-air interface will instead be reflected back into the Au, if the Au film thickness is comparable to or less than their mean free paths (MFPs), the hot electrons will travel back and forth within the Au film before jumping over the Schottky barrier or being relaxed thermally. The maximum number of the round trips of a hot electron depends on the relative energy gained from the photon to Φ_B_ and the Au film thickness, *t*_Au_, to MFP, that is,

5 $$N=\frac{{\rm{MFP}}}{2{t}_{{\rm{Au}}}}\,\mathrm{ln}(\frac{{E}_{{\rm{0}}}}{{{\rm{\Phi }}}_{{\rm{B}}}}),$$

where *t*_Au_ = *t*_top_ or *t*_bot_ for the top or bottom Au film, respectively. Each time the hot electron reaches the Au-TiO_2_ interface, it will have a probability for emission. Therefore, *P*(*E*) in this case is the sum of all the probabilities for all the round trips, as expressed below^27^:

6 $$P(E)=P({E}_{0})+[1-P({E}_{0})]P({E}_{1})+[1-P({E}_{0})][1-P({E}_{1})]P({E}_{2})+\cdots +P({E}_{N})\prod _{m=0}^{N-1}[1-P({E}_{m})],$$

where *E*_i_ = *E*_0_ exp(−2i*t*_Au_/MFP) is the energy of a hot electron after traveling i (=0, 1,…, *N*)-number round trips within the Au film and the corresponding emission probability, *P*(*E*_i_), can be expressed as $P({E}_{{\rm{i}}})=0.5(1-\sqrt{{{\rm{\Phi }}}_{{\rm{B}}}/{E}_{{\rm{i}}}})$ according to Equation (4). Therefore, the more round trips the hot electron experiences, the larger the total IPE probability becomes. If *N* = 0, Equation (6) will reduce to Equation (4) for a one-chance photoemission. In Au, the electron MFP is dependent on the energy gained from the incident light. Here, we set MFP to 70 nm and 18 nm for low (<2 eV) and high (>2 eV) electron energies, respectively^39^. For the solar cell structures shown in Fig. 1, the bottom Au film is 100 nm thick, thicker than the MFP values. The hot electrons gaining solar energy in our considered wavelength range will have only one chance for emission and *P*(*E*) characterized by Equation (4) remains very low. For the top Au film, it includes both flat and curved Au-TiO_2_ contacts. Since the normals at different parts of the curved contacts are all along the radii of the circles as shown in Fig. 1(b), the hot electrons behave the same as those at flat Au-TiO_2_ contacts and therefore *P*(*E*) can be equally characterized by Equations (4) and (6). In this work, the top Au film thickness, *t*_top_, varies from 10 nm to 100 nm and *N* can be large, allowing high *P*(*E*) characterized by Equation (6).

### Current density-Voltage (*J*-*V*) Characterization

Based on the above electronic model and optical simulation, both the top and bottom photocurrent densities, *J*_top_ and *J*_bot_, can be obtained (from Equation (2)) as well as the net photocurrent density, *J*_sc_ = |*J*_top_ − *J*_bot_|, for both polarizations. Considering that the sunlight is typically a natural light with random polarizations, the overall net *J*_sc_ is composed of half-*J*_sc_ for both polarizations. When a bias is applied, the solar cell behaves as a diode and its characteristic *J*-*V* curve is assumed to follow the ideal diode equation for simplicity^6^, i.e.,

7 $$J={J}_{sc}-A\ast {T}^{2}{e}^{-{{\rm{\Phi }}}_{{\rm{B}}}/kT}({e}^{qV/kT}-1)$$

where A* is the effective Richardson constant, *k* is the Boltzmann constant, and *T* = 300 K is the working temperature. From the *J*-*V* curve, *V*_oc_, FF, as well as PCE (=FF·*J*_sc_·*V*_oc_/solar power) can be easily obtained.
